# Supplementary material for: Propagation of goose primordial germ cells in vitro relies on FGF and BMP signalling pathways
Source: Commun Biol. 2025 Feb 25;8:301. doi: 10.1038/s42003-025-07715-7 (PMC11861285; doi:10.1038/s42003-025-07715-7)
Supplement: Supplementary file 2 — Supplementary Information [file 42003_2025_7715_MOESM2_ESM.pdf]

Supplementary Information

Propagation of goose primordial germ cells *in vitro* relies on FGF and BMP signalling pathways

Dadakhalandar Doddamani[1], Bence Lázár[2,3], Kennosuke Ichikawa[1], Tuan-jun Hu[1,4], Lorna Taylor[1], Elen Gócza[3], Eszter Várkonyi[2], Mike J. McGrew[1]

[1]The Roslin Institute and Royal (Dick) School of Veterinary Studies, University of Edinburgh, Easter Bush Campus, United Kingdom

[2]National Centre for Biodiversity and Gene Conservation, Institute for Farm Animal Gene Conservation, Gödöllő, Hungary

[3]Hungarian University of Agriculture and Life Sciences, Institute of Genetics and Biotechnology, Animal Biotechnology Department, Gödöllő, Hungary

[4]National Gene Pool of Waterfowl, Jiangsu Agri-Animal Husbandry Vocational College, Taizhou 225300, China

This file includes:
1 Supplementary Table
9 Supplementary Figures

Supplementary Table 1 Goose medium

A. Goose Medium
Avian KO-DMEM basal medium
- A custom modified version of Gibco Knockout-DMEM (250 mOsm/kg, 12.0 mM glucose, no calcium chloride added) (Thermofisher Scientific #041-96570 M)

Complete Goose Medium without added $CaCl_2$:
47 ml Avian DMEM
1 ml B27 supplement (50x, Gibco 17504044)
0.5 ml Glutamax (100x, Gibco 35050038)
0.5 ml NEAA (100x, Gibco 11140035)
0.5 ml Nucleosides (100x, EmbryoMax ES-008-D)
0.2 ml Pyruvate (100x, Gibco 11360039)
0.1 ml ß-mercaptoethanol (50 mM, Gibco 31350010)
0.5 ml Ovalbumin (20%, Sigma A5503)
0.1ml Na Heparin (50mg/ml, Sigma H3149)
0.1 ml Pen/Strep (100x, Gibco 15070-063)

For 5 mls Goose medium with growth factors:
5ml Compete Goose Medium (no added $CaCl_2$)

- **B12 vitamin: 2.5 µl**
  (stock: 50 mg/ml) (V6629)
- **Cholesterol: 2.5 µl**
  (stock: 2 mg/ml) (Sigma C3045)
- **FGF-1: 4 µl**
  (stock: 10 µg/ml) (R&D Systems 232-FA)
- **FGF-2: 4 µl**
  (stock: 10 µg/ml) (R&D Systems 234-FSE)
- **BMP4: 12.5 µl**
  (stock: 10 µg/ml) (Gibco PHC9534)
- **Ovotransferin: 5 µl**
  (stock: 10 mg/ml) (Sigma C7786)
- **IGF-1: 5 µl**
  (stock: 50 µg/ml) (R&D Systems 291-G1)
- **$CaCl_2$: 3.75 µl**
  (stock: 100 mM)

B. Hungarian Goose medium (50ml) (no retinol and using Commercial DMEM)
Diluted DMEM calcium free medium:
37.5 ml Gibco Calcium free DMEM (Gibco, 21068-028)
11.6 ml Gibco cell culture H2O (Gibco, A12873-01)
0.5 ml Pyruvate (100x, Gibco 11360039)
0.5 ml MEM Vitamin Solution (100x, Gibco 11120052)
0.5 ml MEM Amino acids (Sigma, M5550)

Compete Goose Medium without added $CaCl_2$:
47 ml Diluted DMEM Calcium free medium
1 ml B27 supplement (50x, Gibco 17504044)
0.5 ml Glutamax (100x, Gibco 35050038)
0.5 ml NEAA (100x, Gibco 11140035)
0.5 ml Nucleosides (100x, EmbryoMax ES-008-D)
0.2 ml Pyruvate (100x, Gibco 11360039)
0.1 ml ß-mercaptoethanol (50 mM, Gibco 31350010)
0.5 ml Ovalbumin (20%, Sigma A5503)
0.1ml Na Heparin (50mg/ml, Sigma H3149)
0.1 ml Pen/Strep (100x, Gibco 15070-063)

For 5 mls Goose medium:
5ml compete Basal Medium without added $CaCl_2$

- **B12 vitamin: 2.5 µl**
  (stock: 50 mg/ml) (Sigma V2876)
- **Cholesterol: 2.5 µl**
  (stock: 2 mg/ml) (Sigma C3045)
- **FGF-1: 4 µl**
  (stock: 10 µg/ml) (Thermo Fisher 13241013)
- **FGF-2: 4 µl**
  (stock: 10 µg/ml) (Thermo Fisher 13256-029)
- **BMP4: 12.5 µl**
  (stock: 10 µg/ml) (Gibco PHC9534)
- **Ovotransferin: 25 µl**
  (stock: 10 mg/ml) (Sigma C7786)
- **IGF-1: 5 µl**
  (stock: 50 µg/ml) (Thermo Fisher RP-10931)

- **$CaCl_2$: 3.75 µl**
  (stock: 100 mM) (Sigma C4901)

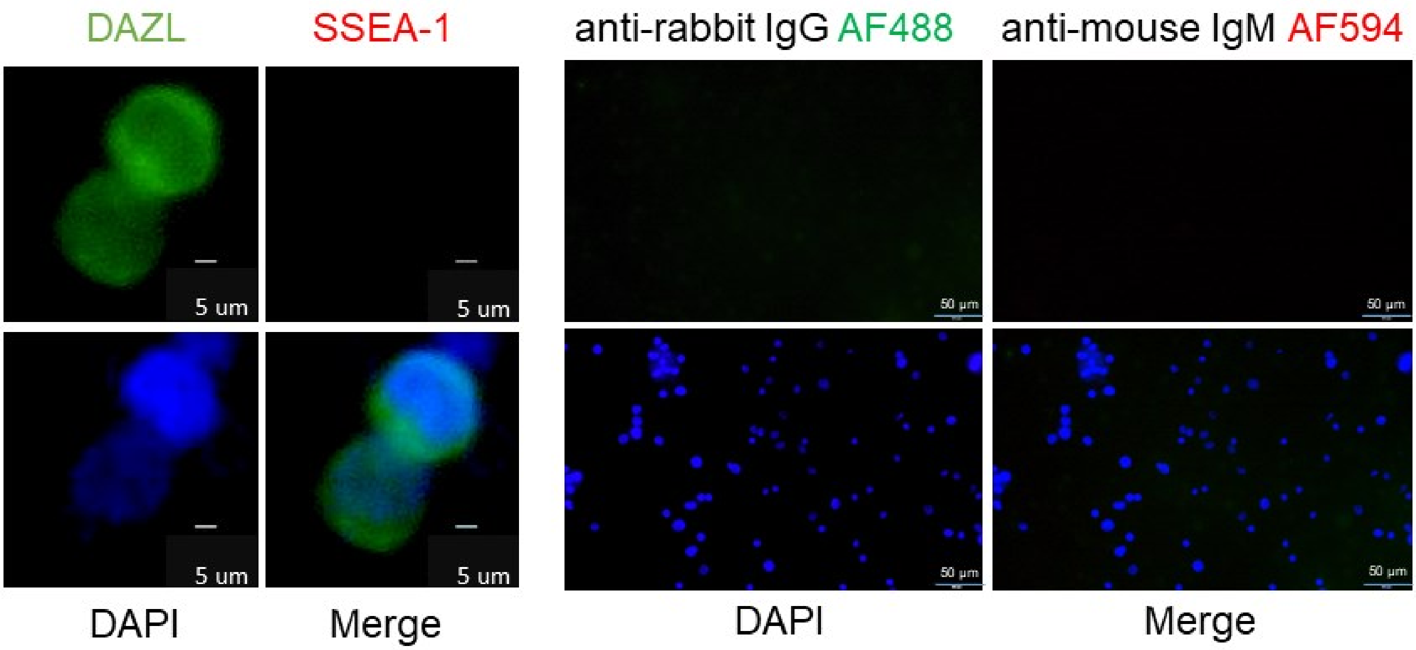

Supplementary Figure 1: Immunostaining of Goose PGCs.
Male goose PGCs stained with DAZL and SSEA1 antibodies. The no primary control staining (right images) indicates that the DAZL cytoplasmic staining is specific for DAZL protein.

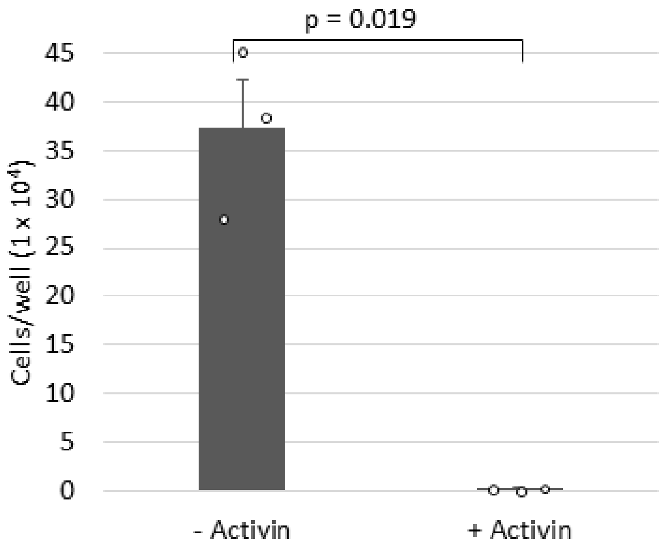

Supplementary Figure 2: Proliferation of goose PGCs with or without additional Activin A. The addition of Activin A inhibits cell proliferation significantly.

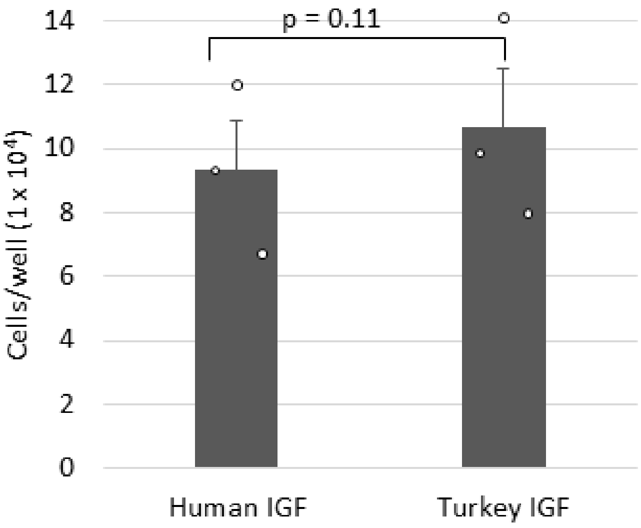

Supplementary Figure 3: Proliferation of goose PGCs with avian or mammalian IGF-1. There is no significant difference in cell proliferation either using human-derived or turkey-derived IGFs.

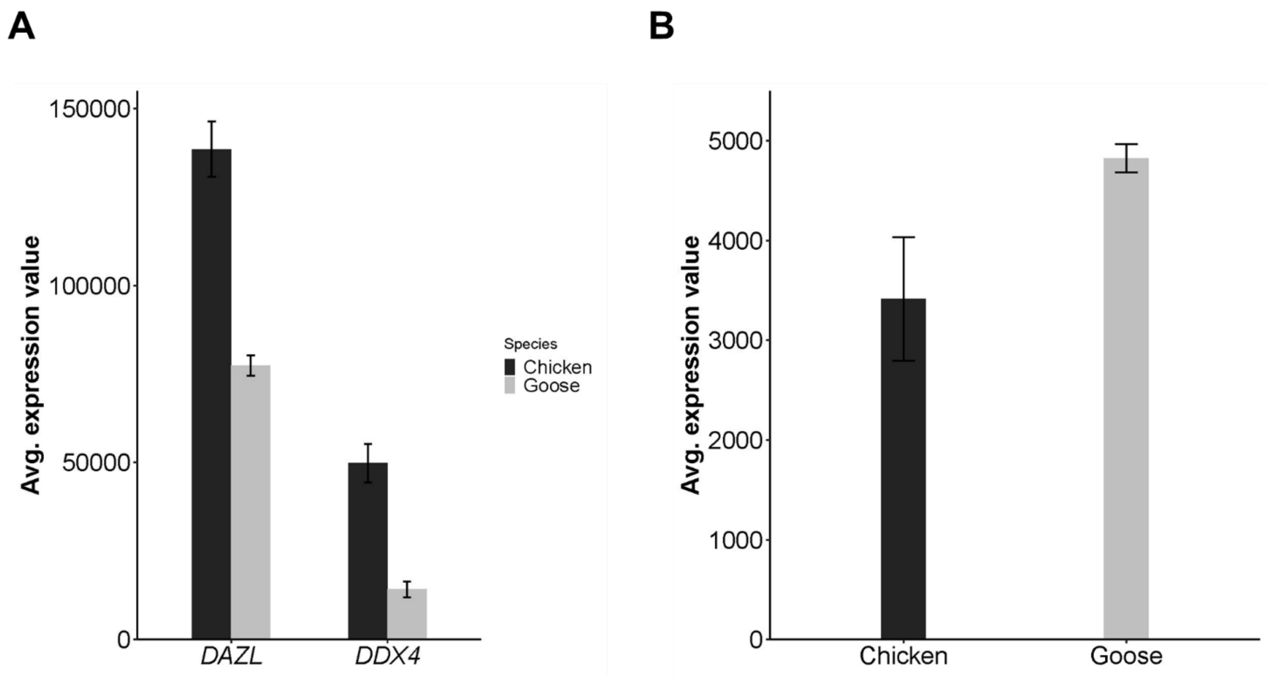

Supplementary Figure 4: RNA transcriptome expression comparison between chicken and goose PGCs. **A.** Known avian germ cell-specific markers like *DAZL* and *DDX4* are expressed in the goose PGCs. **B.** The expression of the pluripotent gene *PRDM14* is conserved in goose PGCs.

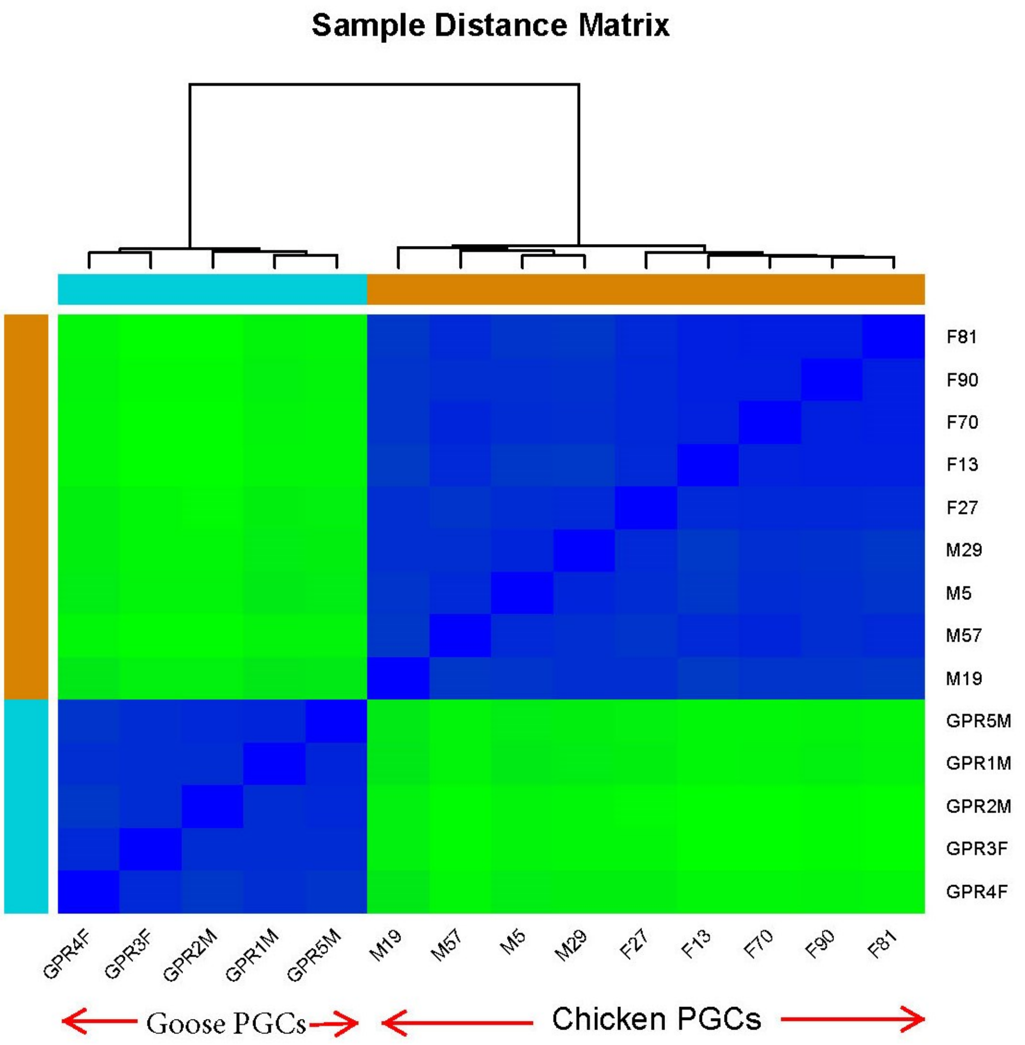

Supplementary Figure 5: Heatmap showing the distance matrix based on the transcriptome profile of chicken and goose PGCs. Brown, chicken PGC lines, Green, goose PGC lines. M, F indicates male or female PGC line.

A

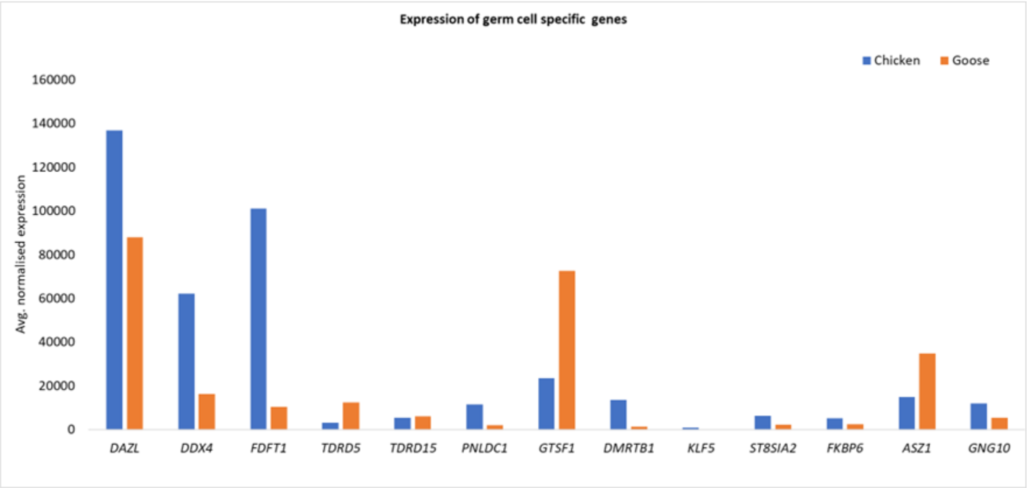

B

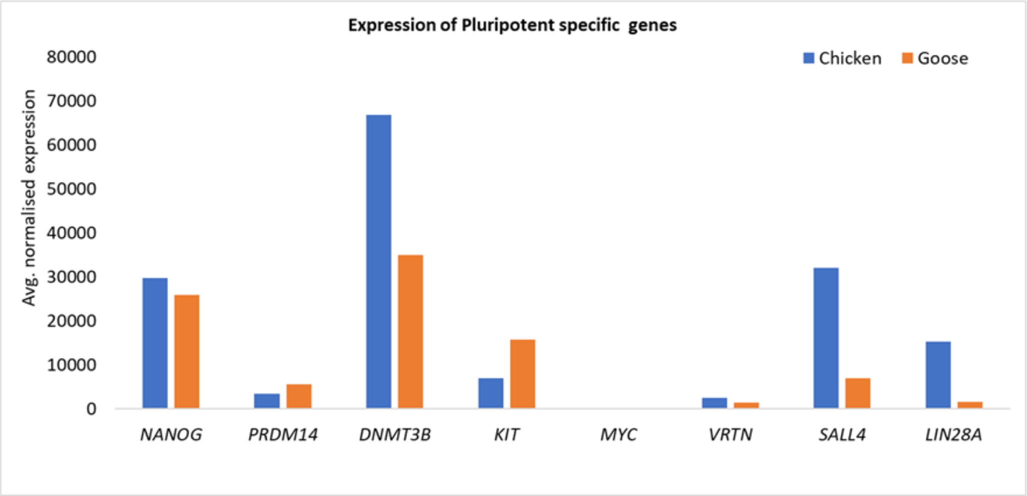

Supplementary Figure 6: RNA transcriptome analysis of genes expressed specifically in chicken and goose PGCs. The conserved expression profiles of germ cell-specific markers (A) and pluripotent markers (B) in chicken and goose PGCs.

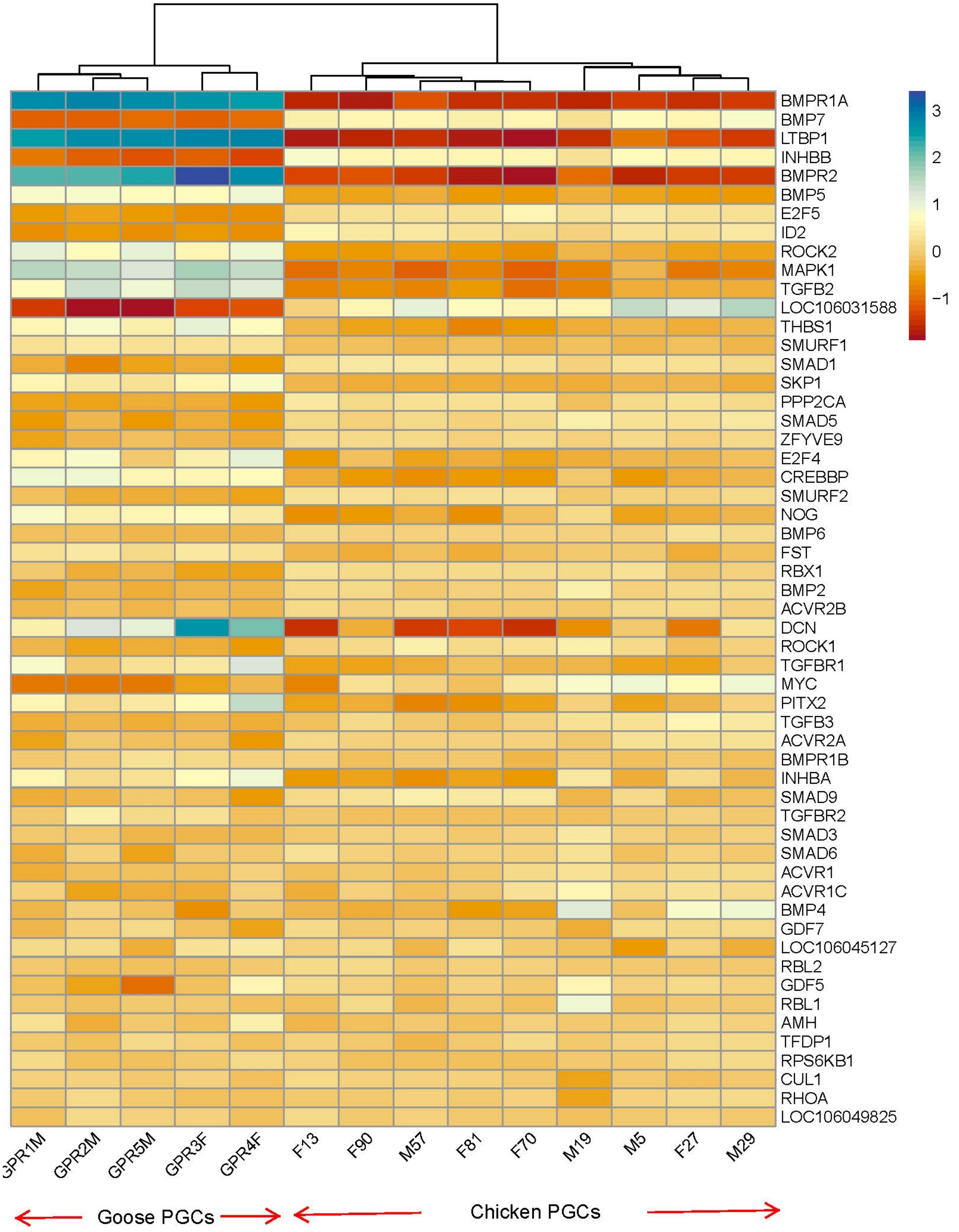

Supplementary Figure 7: Heatmap showing the expression pattern of TGF-β signalling-related genes in chicken and goose PGCs. The change in expression of a few genes shows the TGF-β signalling pathway is different in these species. However, it should be noted that chicken PGCs and goose PGCs are grown in FAOT and FBOT medium, respectively.

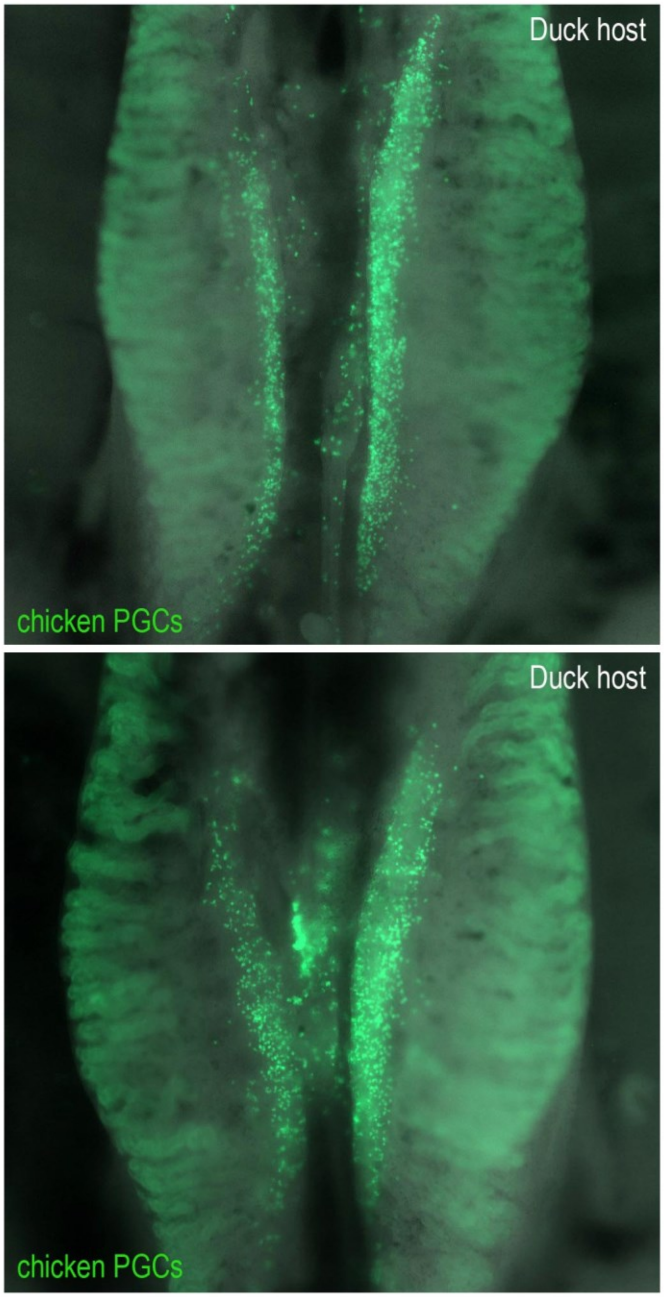

Supplementary Figure 8: Duck embryos injected with chicken PGCs

Duck embryos were injected with cultured GFP^{+} chicken PGCs and incubated for 4 days then imaged for GFP fluorescence.

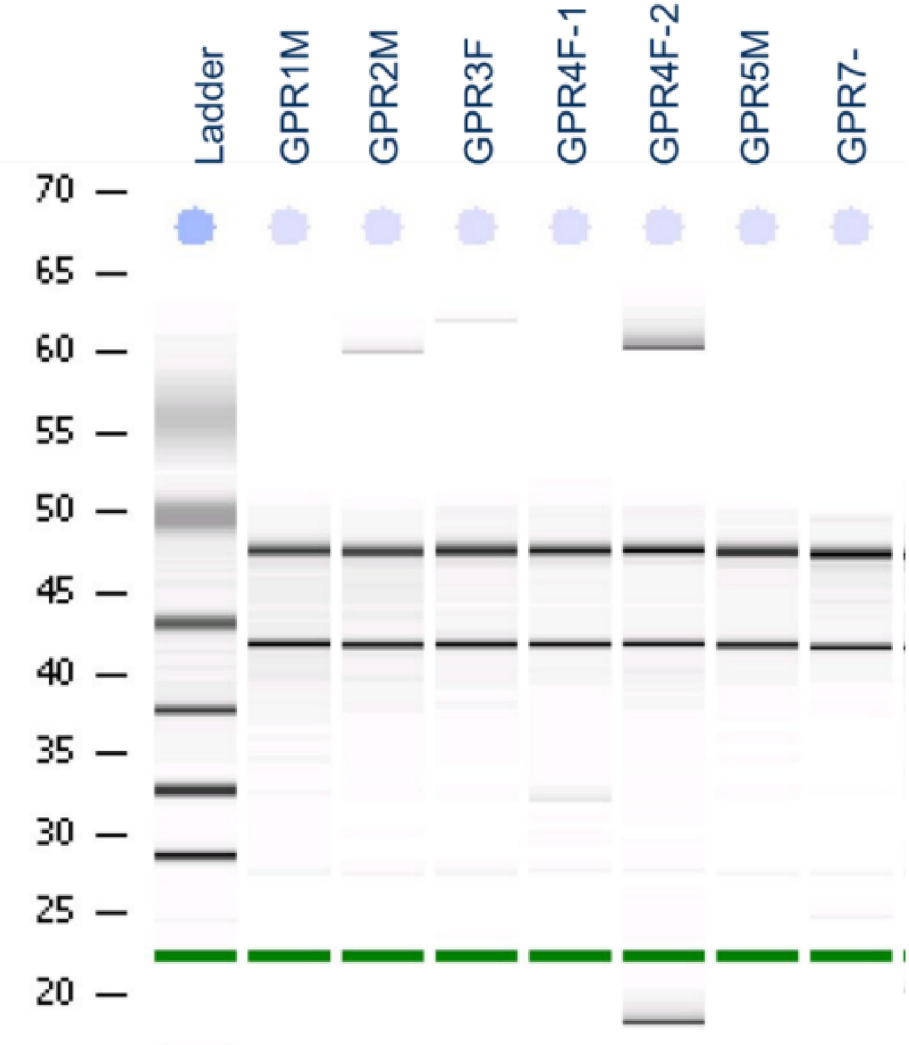

Supplementary Figure 9: Bioanalyzer gel-like image showing the quality of the total RNA from the goose PGCs. Distinct ribosomal RNA bands 28S and 18S found for the total RNA isolated from these samples indicate good-quality intact RNA.
