# Supplementary material for: Propagation of goose primordial germ cells in vitro relies on FGF and BMP signalling pathways
Source: Commun Biol. 2025 Feb 25;8:301. doi: 10.1038/s42003-025-07715-7 (PMC11861285; doi:10.1038/s42003-025-07715-7)
Supplement: Supplementary file 3 — Description of Additional Supplementary Files [file 42003_2025_7715_MOESM3_ESM.pdf]

Description of Additional Supplementary Files

File name: Supplementary Data 1

Description: Numerical source data for all graphs and analyses in the manuscript

File Name: Supplementary Data 2-5

Description: Supplementary Data Tables 2-5
